# Evaluating the Feasibility of an Agglomerative Hierarchy Clustering Algorithm for the Automatic Detection of the Arterial Input Function Using DSC-MRI

**DOI:** 10.1371/journal.pone.0100308

**Published:** 2014-06-16

**Authors:** Jiandong Yin, Jiawen Yang, Qiyong Guo

**Affiliations:** Department of Radiology, Shengjing Hospital of China Medical University, Shenyang, Liaoning, China; Aarhus University, Denmark

## Abstract

During dynamic susceptibility contrast-magnetic resonance imaging (DSC-MRI), it has been demonstrated that the arterial input function (AIF) can be obtained using fuzzy c-means (FCM) and *k*-means clustering methods. However, due to the dependence on the initial centers of clusters, both clustering methods have poor reproducibility between the calculation and recalculation steps. To address this problem, the present study developed an alternative clustering technique based on the agglomerative hierarchy (AH) method for AIF determination. The performance of AH method was evaluated using simulated data and clinical data based on comparisons with the two previously demonstrated clustering-based methods in terms of the detection accuracy, calculation reproducibility, and computational complexity. The statistical analysis demonstrated that, at the cost of a significantly longer execution time, AH method obtained AIFs more in line with the expected AIF, and it was perfectly reproducible at different time points. In our opinion, the disadvantage of AH method in terms of the execution time can be alleviated by introducing a professional high-performance workstation. The findings of this study support the feasibility of using AH clustering method for detecting the AIF automatically.

## Introduction

Cerebral perfusion describes the steady-state delivery of nutrients and oxygen via blood to the brain tissue parenchyma, and it comprises several cerebral hemodynamic parameters, i.e., cerebral blood flow (CBF), cerebral blood volume (CBV), and mean transit time (MTT), which play important roles in the diagnosis and management of many diseases [1]–[5]. At present, several imaging techniques are used to analyze cerebral perfusion, including positron emission tomography, single photon emission computed tomography, and CT. However, most have the disadvantage of using ionizing radiation. By contrast, magnetic resonance imaging (MRI) does not have this shortcoming [6]–[9]. The technique of dynamic susceptibility contrast (DSC) using an intravascular contrast agent is applied most frequently to the quantification of cerebral hemodynamics [10]. This method monitors the signal changes induced by the paramagnetic contrast agent as it passes through cerebral vessels and a series of T2- or T2*-weighted images are acquired over time [7], [11], [16].

According to indicator dilution theory, the quantification of cerebral hemodynamics using the DSC-MRI technique requires that these tissue voxel time courses are corrected for the tracer concentration in an artery that feeds the corresponding part of the brain, i.e., arterial input function (AIF) [12], [13].In particular, to evaluate the CBV, the tissue concentration curves are normalized by the time integral of AIF [14], [15], i.e.,
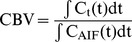
, and the tissue concentration curve are deconvolved with AIF to evaluate the CBF, as follows [2], [6], [15], [16]:
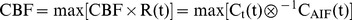
(1)where 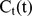 is the time-concentration curve of the contrast agent in the tissue voxel of interest (VOI), 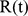 is the fraction of the injected tracer still present in the vascular at time t after an infinitely short injection of a tracer, 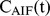 is the AIF, and 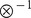 represents a deconvolution operation, which is highly sensitive to the AIF shape [14].

Thus, it is very important to obtain a reliable AIF before the subsequent quantification of physiologically relevant parameters [14]. The original methods for AIF detection were based on the manual extraction of a region of interest, such as the middle cerebral artery (MCA) and internal carotid artery (ICA), which depended on the user's experience and subjective judgments, thereby leading to low accuracy and consistency [1], [8], [17]–[19]. Thus, the development of an automatic AIF detection method has been an interesting and demanding research problem. To identify AIF automatically, Murase *et al.* and Mouridsen *et al.* proposed techniques based on fuzzy *c*-means (FCM) and *k*-means clustering algorithms, respectively [8], [18]. The feasibility of these two clustering algorithms for AIF detection was validated, but both share the drawback that the analysis results are reliant on the initialization of cluster centers, which result in poor reproducibility at repetition of AIF measurements.

The present study proposes a clustering method based on the agglomerative hierarchy (AH) algorithm, which was described briefly in the later section of present manuscript. The performance of the AH method was compared with previously reported methods using FCM and *k*-means clustering in terms of detection accuracy, computational complexity, and calculation reproducibility. Computer simulation as well as clinical study was performed to evaluate the feasibility of the novel method for AIF detection.

## Materials and Methods

All of the calculations and simulations were performed using an off-line personal computer (Inter Core i3 M350 CPU processor, 2.27 GHz operating frequency, 2.0 GB RAM memory capacity, Microsoft Windows 7 operating system). A MATLAB program was developed in our department for AIF detection.

### 1. Clinical DSC-MRI perfusion data

Ethical clearance for this study was obtained from the Ethics Committee at Shengjing Hospital, China Medical University (No. 2013PS113K), and written informed consent was obtained from each participant before the study commenced.

Clinical DSC-MRI perfusion data were obtained from 42 healthy volunteers (aged, 23–69 years; average age, 49.5 years; weight, 58±14 kg; 27 males and 15 females). DSC-MRI acquisition was performed using a 3.0 T whole-body MR scanner with multichannel capabilities (MAGNETOM Verio; Siemens Medical Solution, Erlangen, Germany). Perfusion imaging was performed using a single-shot echo planar imaging (EPI) sequence with the following parameters: TR  = 1500 ms, TE  = 30 ms, matrix  = 128×128, field of view (FOV)  = 23×23 cm, slice thickness  = 4 mm, spacing between slices  = 5.2 mm, slice number  = 19, acquisition type  = 2D, number of phase-encoding steps  = 127, transmitting coil  =  body, and flip angle  = 90°.

At the seventh time point, a gadolinium dose of 0.1∼0.2 mmol/kg (Gadovist; Bayer Schering Pharma AG, Berlin, Germany) was administered, followed by an equal volume of saline flush, which were both delivered at 4 ml/s. The horizontal part of the right MCA was crossed by an imaging slice. Sixty-two whole-head images were obtained (scanning time  = 93 s) per subject. The magnetization state was not steady at the beginning of perfusion scanning, so the first two images were discarded, and time 0 was assigned to the third volume acquired. Therefore, 60 volumes were used for subsequent analysis.

### 2. Simulation scheme

To assess the feasibility of AH clustering method for AIF detection, a simulation study was also conducted in the same manner proposed by Peruzzo *et al.* [1] and Wu *et al.* [21], where a ‘true’ and a ‘false’ AIF was modeled. Partial volume effect (PVE) was simulated using three kinds of tissues, normal gray matter (GM), normal white matter (WM) and pathological GM. Typical noise was added.

#### 2.1 Simulated AIF

The AIF was modeled using the following equation, which presented the shape and size obtained by a standard contrast agent injection scheme [8], [16].



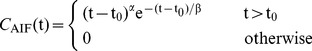
(2)where 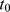 represents the tracer arrival time,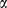 is the measure of inflow velocity steepness, and 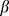 is the washout velocity. A recirculation process was added that comprised a copy of the aforementioned function with a delay of 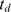, which was convolved with an exponential with a time constant of 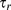. We used values of 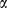 = 3.0, 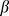 = 1.5 s, 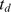  = 8 s, 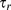  = 30 s [1], [6], [8], [16], and 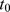  = 26 s, which approximated the arrival time of the contrast agent in our clinical perfusion data.

#### 2.2 Simulated tissue signal

The residue function 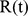 was modeled using a gamma-variate function, as follows:
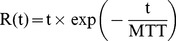
(3)where 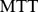 was calculated as 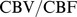 according to the central volume theorem.

The concentration function of the contrast agent in a tissue voxel 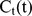 was calculated using inversion formula of Eq. 1.

The concentration of the contrast agent was converted into the MRI signal intensity using the following equation [15], [16], [21], [22] under the assumption that Ct is proportional to Delta R2*:
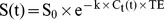
(4)where 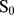  = 100 is the baseline signal intensity, 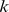 is a constant that causes a 40% signal peak decrease from baseline with normal gray matter (GM) after the tracer injection, which corresponds to the values typically found in clinical cases, and TE is the echo time of the scanning sequence. The signal time-intensity curves were generated by simulating our real clinical perfusion data, where the scanning duration was 90 s and TE  = 30 ms.A sampling rate of 1s was used.

Six true arterial voxels were included in the simulation study and 16 false arterial voxels were simulated by varying 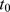 from 27 to 30 s, and 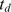 from 9 to 12 s in increments of 1.0 s. In addition, 440 voxels representing normal GM tissue were simulated by setting CBV  = 4 ml/100 g, MTT  = 4±0.33 s; 440 voxels that represented pathological GM tissue were simulated by setting CBV  = 3.3 ml/100 g, MTT  = 10±0.7 s;600 voxels that represented normal white matter tissue were simulated by setting CBV  = 2 ml/100 g, MTT  = 5.45±0.33 s; and 400 voxels contaminated by the PVE were simulated using linear combinations of a true arterial signal and a signal for one of the different tissues, where the weights were selected at random.

Finally, noise was added to 100 randomly extracted curves using a previously reported technique [1], [16]. Noise was modeled as a zero-mean Gaussian function where the standard deviation (SD) was selected to produce a signal-to-noise ratio (i.e., SNR  =  S_0_/SD) of 20 as well as 40 and 60.

### 3. AIF calculation

#### 3.1 AIF determination using simulated data

The AIFs were calculated from the time-intensity curves of the simulated signals as follows.

First, the signal intensity curves were converted into tracer concentration curves, according to the following equation [6], [7], [18]:
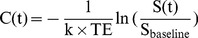
(5)where 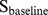 was the baseline signal intensity (i.e. t <26 s), which was obtained by averaging the pre-contrast signal. Next, two previously used methods, i.e., *k*-means and FCM, were applied to the converted data according to the mathematical principles outlined in Refs 8 and 18, in which the parameter setting methods for AIF detection are also described. The description of the application of AH clustering is described briefly in the following.

The time-concentration curves that need to be clustered are treated as a set of N items and the pairwise distance or similarity between each two of the input vectors is calculated using the Euclidean distance.

The iterative process of AH clustering is executed as follows.

1. Assign each item to a cluster to obtain N initial clusters.
2. Find the closest pair of clusters (shortest distance) and merge them into a single cluster.
3. Calculate the distance between each two of the new clusters. The distance can be computed using three different methods: single-linkage, complete-linkage, and average-linkage. In the present study, we apply average-linkage clustering, i.e., the distance between one cluster and another cluster is considered to be equal to the average distance between any member of one cluster and any member of the other cluster.
4. Steps 2 and 3 are repeated until the desired number of clusters is obtained.

As described in previous studies [1], [8], [18], the clustering number was set to five. In general, the tracer time-concentration curves in the arteries are characterized by a higher maximum concentration, an earlier maximum concentration, and a narrower full-width half maximum (FWHM), which allows the arterial curves to be distinguished from venous curves that appear wider with a later bolus arrival, and tissue curves that are wider with a lower peak height [1], [7], [17], [18]. Thus, in order to determine which cluster best represented the AIF automatically, several parameters related to the mean curve of each cluster were calculated, such as the maximum concentration (peak height, H_P_), time of maximum concentration (time to peak height, T_P_), and FWHM, as well as a measure (M) given by H_P_/[T_P_×FWHM]. The cluster with the maximal M value was considered to contain arterial pixels and the AIF was obtained as the mean curve [8].

#### 3.2 AIF determination using clinical data

**Figure 1:**
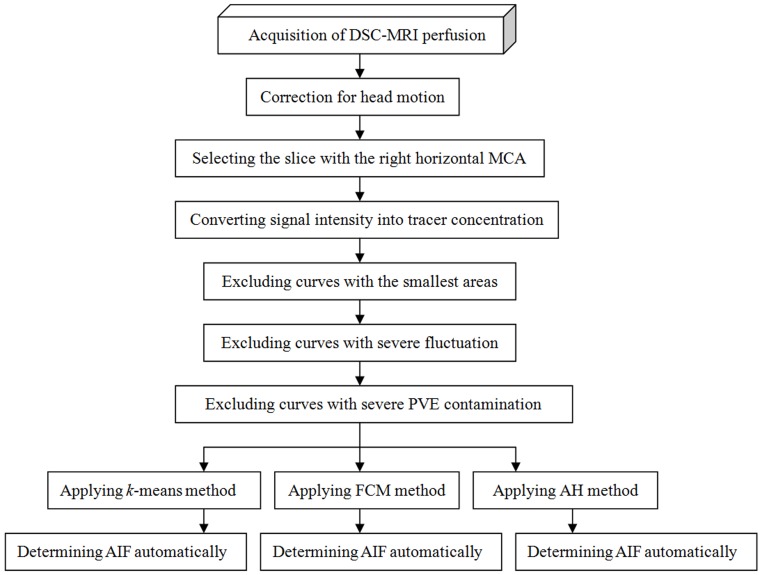
Flowchart showing the automatic AIF detection processes applied to clinical DSC-MRI perfusion data in the present study.

Fig. 1 shows a flowchart that illustrates the automatic AIF detection process applied to clinical perfusion data. The details of this process are as follows.

First, due to the misalignments among the volume images at different scanning time points caused by physiological fluctuations (such as breathing and heartbeats) and the involuntary motions of the subjects, which could corrupt the shape of the first passage and result in serious quantification errors, all of the volumes were aligned to the first pre-contrast volume using two well-known software packages: SPM(http://www.fil.ion.ucl.ac.uk/spm/ *)* (version, SPM99)and INRIAlign 1.01 (http://www-sop.inria.fr/epidaure/Collaborations/IRMf/INRIAlign.html) [23]–[25]. Image smoothing can lead to bias during hemodynamic quantification, so no smoothing operations were performed on any of the images.

Second, the slice image containing the right horizontal MCA was extracted from the first dynamic volume image. The selection of an optimal slice affects the accuracy of AIF determination. Previous reports have shown that selecting a slice containing the MCA causes fewer hemodynamic quantification errors than a slice containing the ICA because the delay and dispersion are minimized between the AIF measurement location and peripheral tissue [1], [12], [13], [26]. Eq. (5) was applied to the given slice to convert the signal time-intensity curves into tracer time-concentration curves.

Third, only a small fraction of the entire set of time-concentration curves represented arteries. Most of the curves correspond to other tissue voxels where the curves changed very slightly. It is necessary to eliminate these weak pixels to optimize AIF detection. Thus, we computed the area under each curve (AUC) and excluded the P_AUC_ percentage of curves with the smallest areas [18].

Fourth, some irregular and distortion time-concentration curves due to PVE bias, shifts in voxels, and physiological pulsations were obtained during clinical data scanning. These rough and erratic curves would lead to poor estimates of the true AIF. Therefore, the following measure was used and the P_Rough_ percentage of the curves with the largest integral values was discarded [18].
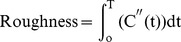
(6)


Based on Mouridsen *et al*., the values of P_AUC_ and P_Rough_ were set to 0.90 and 0.25, respectively [18].

Fifth, the new criterion proposed by Bleeker et al. was utilized to further reduce the effects of PVE on the AIF measurement [17], [26], which relies on the fact that the perfusion parameters could be measured from the second passage as well as the first passage. The new guidelines for the detection of PVE was carried out by using the ratio of the steady-state integral value relative to the AUC of the first passage, which should result in an equal value for tissue and arterial responses. It should be emphasized that this criterion was simplified in the present study. First, the first tracer passage was fitted using a Gamma-variate function [27]. The area under the fitted curves was calculated and abbreviated to AUC^1st^. Second, the start point of the steady state was assigned to the first time point after the peak that was <30% of the maximum of the time-concentration curve [28]. The 10 succeeding time points were then integrated and the result was abbreviated to AUC^2nd^ [17]. Finally, the ratio of AUC^2nd^ to AUC^1st^was calculated for all of the remaining curves, and curves with ratios that fell outside the range of acceptance (mean ratio ±20%) were considered to be severely contaminated by PVE and excluded [17].

Finally, AH, FCM, and *k*-means clustering algorithms were applied separately to the residual curves. The clustering number was still set to five and the AIF curve was determined automatically using the measure M  =  H_P_/[T_P_×FWHM] once again.

### 4. Statistical analysis

The performance of the AH clustering method was evaluated based on comparisons with the traditional *k*-means and FCM algorithms, which are currently applied methods for AIF detection.

#### 4.1 Statistical analysis of simulated data

Statistical analyses were performed using various shape parameters, such as the FWHM, T_P_, and H_P_, to evaluate the AIF detection accuracy [29]. Moreover, the PVE level was defined as the percentage of non-arterial curves in clusters selected as arterial curves [1]. A low PVE level indicated that the corresponding algorithm performed well in distinguishing arterial voxels from other tissue elements. In general, an estimated AIF with a lower PVE level had a greater H_P_ and a smaller FWHM, while an earlier T_P_ indicated that the relevant algorithm was affected less by bolus delay and dispersion [29], [30].

Second, the quantification of CBV depended on the AIF integral, so the AUC was used as another important parameter to assess the estimated AIF [1], [29].

Finally, the difference between the estimated AIF and the true AIF was computed as the root mean square error(RMSE):
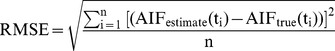
(7)where n was the scanning duration (90 s).

#### 4.2 Statistical analysis of in vivo measurements

First, in the same manner as the simulation study, the accuracy of AIF detection using the three different clustering methods was assessed based on shape parameters (H_P_, T_P_, and FWHM), M values, and AUCs.

Second, the calculation-recalculation reproducibility of each method was evaluated by comparing the AIF results calculated independently 100 times in succession. The robustness, which is defined as the variance of AIF curves, was quantified using:
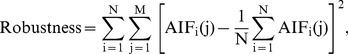
(8)where M  = 60 represents the number of dynamic scanning volumes and N = 100 is the number of repeated calculations [20].

Third, the calculation time for each clustering method was recorded to determine the algorithm that could perform AIF selection most rapidly.

The statistical analysis was performed using SPSS (SigmaStat, 2.03, Inc., Chicago, IL). The difference was investigated using a paired-samples *t*-test, where *P*<0.05 was considered significant.

## Results

### 1. Results of the simulation study

**Figure 2:**
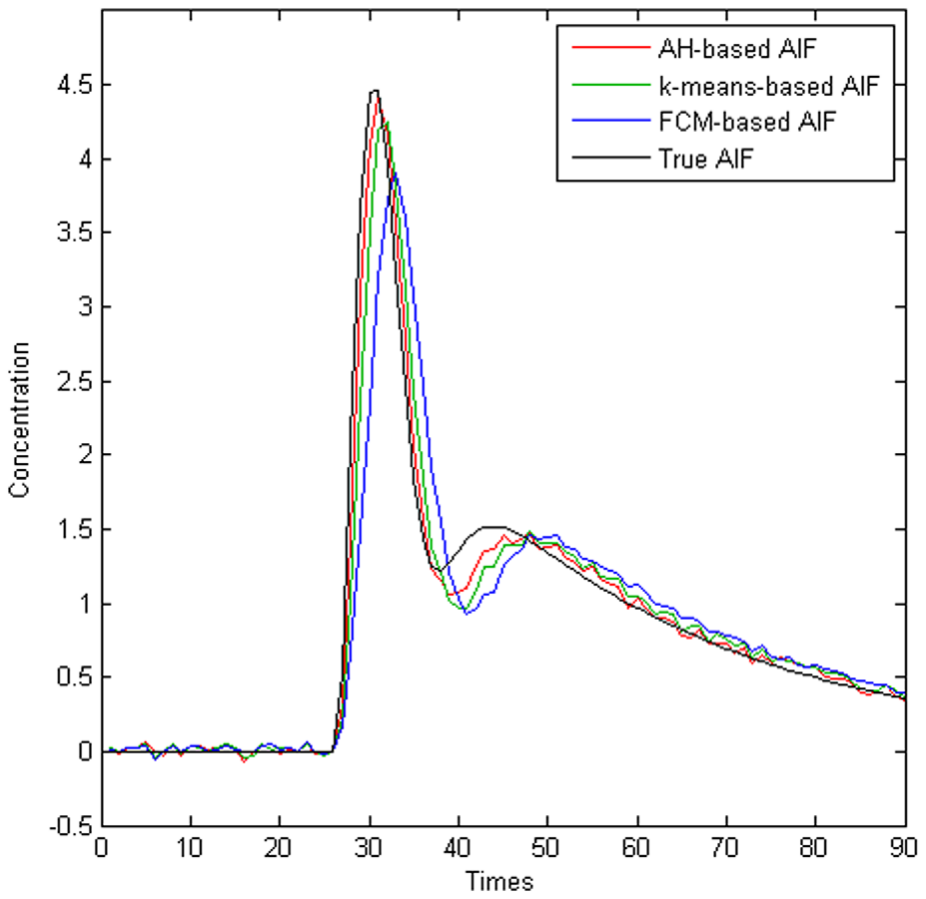
Comparison of AIFs obtained using different clustering algorithms and the true AIF.

The results of the simulation study are shown in Fig. 2 and Table 1. Fig. 2 shows a comparison of the estimated AIFs derived from the three clustering methods and the true AIF when SNR  = 20. Table 1 shows the results of the statistical analysis.

**Table 1:** Comparison of AIFs obtained using different clustering methods and the true AIF.

SNR	AIF	PVE	H_P_	T_P_	FWHM	AUC	RMSE	M
20	FCM-based AIF	0.7273	3.9101	31.87	7.3487	75.8796	0.4647	0.0167
	*k*-means-based AIF	0.5714	4.3001	30.60	6.6031	76.1378	0.2628	0.0213
	AH-based AIF	0.4000	4.4030	30.01	6.3188	76.2088	0.1374	0.0232
40	FCM-based AIF	0.7143	3.9217	31.54	7.2636	75.9328	0.4463	0.0173
	*k*-means-based AIF	0.5385	4.3561	30.52	6.4002	76.2001	0.2302	0.0218
	AH-based AIF	0.3333	4.4436	29.99	6.2283	76.4711	0.1317	0.0239
60	FCM-based AIF	0.7000	4.0726	31.23	7.1103	76.1017	0.4028	0.0197
	*k*-means-based AIF	0.5000	4.3982	30.37	6.3904	76.3164	0.2077	0.0220
	AH-based AIF	0.3333	4.4436	29.99	6.2283	76.4711	0.1317	0.0239
0	True AIF	0	4.5369	29.51	6.2016	76.8679	0	0.0247

Based on a visual inspection of Fig. 2, we can see that AH-based method produced the nearest approximation to the true AIF and this conclusion was validated quantitatively by the RMSE indicator shown in Table 1. Table 1 also shows that the AH-based AIF was affected least by PVE contamination. Compared with the two previously reported clustering methods, AH clustering method also produced a larger AUC, higher peak, narrower FWHM, and a lower trend line at the tail, which also demonstrate that AH method was affected less by PVE during AIF detection [29]–[31].Moreover, T_P_ occurred earlier with AH clustering method than FCM and *k*-means algorithms, which shows that AH method was affected less by tracer transport delays [30]. Table 1 also shows that the M value of AH-based AIF was much closer to that of the true AIF than both *k*-means-based and FCM-based AIFs.

### 2. Results of the clinical verification

**Figure 3:**
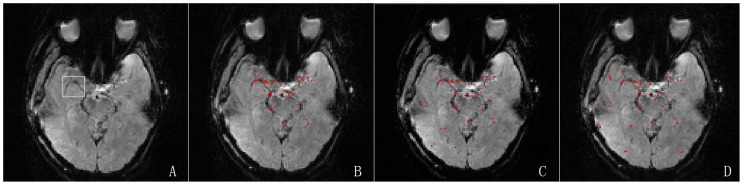
Results of AIF detection based on different clustering methods. The first sub-figure showed the optimal slice image of the randomly selected subject for clustering analysis which was extracted from the first volume of a series of dynamic images, and the right MCA was highlighted by the white rectangle. Sub-fig. B, C and D illustrated the results obtained using AH, *k*-means and FCM clustering where the red points represented the arterial areas.

**Figure 4:**
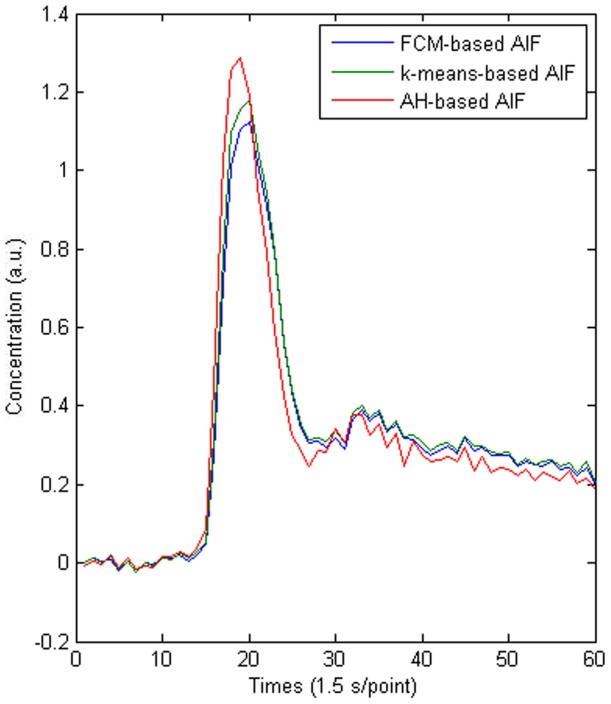
Comparison of the AIFs obtained using *k*-means, FCM, and AH clustering methods.

According to the processes described earlier, separate AIFs were estimated by applying the three clustering methods to each participant. A randomly selected subject (a 48-year-old female) is used to illustrate the AIF detection performance of the different algorithms (Figs 3 and 4). Figure 3 shows the same regions and the different regions that were selected as arterial voxels using the three different clustering methods. Similar arterial regions were extracted and only minor differences were visible. Figure 4 shows that compared with AH-based AIF, the AIFs determined using the *k*-means and FCM methods were similar to those in the simulation study, and they had smaller peaks and higher tails.

The statistical results for the 42 volunteers are shown in Table 2. Table 2 indicates that the results of the clinical study had similar trends to the simulation study, i.e., a higher H_P_, narrower FWHM, earlier T_P_, larger AUC, and larger M values for AH clustering compared with both FCM and *k*-means clustering methods. The statistical result demonstrated that the H_P_ of AIF based on AH clustering method was significantly different compared with those based on both FCM and *k*-means algorithms (*P*<0.05). There was no significant difference in T_P_ between AH and FCM methods, and between AH and *k*-means methods (*P*>0.05). The difference in FWHM was significant between AH and FCM methods (*P*<0.05),but not between AH and *k*-means clustering methods (*P*>0.05).There were significant differences in both the AUC and M values between AH and FCM clustering, and between AH and *k*-means clustering (*P*<0.05).These results indicate that AH algorithm can obtain a more accurate AIF than each of the other two methods [29], [30].

**Table 2:** Comparison of the AIFs obtained using different clustering methods.

Methods	Shape parameters			AUC (Mean±SD)	M value (Mean±SD)	Time required (s) (Mean±SD)	Robustness (Mean±SD)
	H_P_ (a.u.) (Mean±SD)	T_P_ (s) (Mean±SD)	FWHM (s) (Mean±SD)				
FCM	1.1911±0.2679	32.61±1.90	7.3034±0.6839	16.7885±1.2487	0.0093±0.0012	0.163±0.0689	8.9735±2.0942
*k*-means	1.3762±0.3690	31.20±1.55	7.0621±0.7825	18.1993±2.8734	0.0117±0.0014	0.297±0.08936	1.3843±0.8327
AH	1.6896±0.1768	30.55±1.09	6.3165±0.8846	18.9324±1.2356	0.0142±0.0011	393.438±68.368	0.0000±0.0000

Table 2 shows clearly that the execution times varied enormously between AH and the other two methods, and the difference was significant (*P*<0.05). Thus, high computational complexity was an undesirable drawback of AH method during AIF determination.

**Figure 5:**
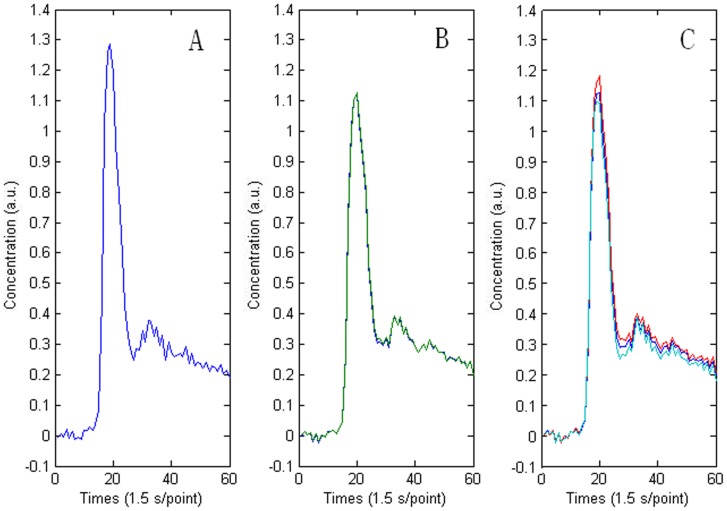
Comparison of reproducibility of the AIF detection using AH (A), FCM (B), and *k*-means (C) clustering methods. Each algorithm was executed independently 100 times in succession, and the robustness values were respectively 0.0000 for AH, 0.0032 for FCM, and 0.0007 for *k*-means.

In addition, the calculation reproducibility of each AIF detection method was addressed. Each algorithm was executed 100 times independently. Table 2 shows that the robustness indicator was zero with AH method but greater than zero with FCM and *k*-means methods, which demonstrates that the AIFs were perfectly reproducible with AH algorithm but not with FCM and *k*-means methods. For the randomly selected example, the same conclusion can be derived intuitively from Fig. 5.

## Discussion

The quantification of cerebral perfusion can provide important information during clinical diagnosis and treatment of several pathologies related to cerebral hemodynamics. However, DSC-MRI quantification requires advance knowledge of the AIF. The conventional manual selection method for AIF is time-consuming and experience-dependent, which results in irreproducible AIF detection results [1], [17]. Thus, an automatic and accurate method for AIF detection is essential in routine clinical practice.

In this study, we developed a novel approach based on AH clustering to measure the AIF. This method was verified using both simulated and clinical data based on comparisons with two previously reported methods for AIF detection, i.e., *k*-means and FCM clustering techniques, in term of detection accuracy, computational complexity, and calculation-recalculation reproducibility. The true AIF was present in the simulated section and we conducted a comparison to determine the algorithm that performed the best at AIF selection. Figure 2 shows that AH-based AIF obtained results that were closer to the true AIF than the FCM- and *k*-means-based AIFs. Table 1 also shows that the parameters derived from AH-AIF were closer to those for the true AIF. Thus, the AIF detection performance of AH clustering was better than that of the FCM and *k*-means methods. Similar results were obtained in the clinical study. Compared with other tissue curves, it is expected that the AIF curves represent the shape features with a higher H_P_, narrower FWHM, lower PVE, earlier T_P_, and larger AUC, etc. Hence, AH clustering yielded AIFs more in line with the expected AIF [30], [31]. Maybe this is dependent on two good qualities of AH method, clustering analysis and iterative procedure [1]. First, cluster analysis can select nodes with similar kinetics, as it is based on Euclidean distance computed for whole time-concentration curves. Thus, an increased robustness against noise of AH method is achieved, since all noisy voxels have different kinetics. Second, the iterative approach is another advantage of the AH clustering method, which differs from *k*-means and FCM clustering. In this study, although *k*-means and FCM clustering methods used the same cluster analysis, it performed only two processing steps, which might result in the consequence that they were more influenced by PVE as shown in Table 1. Compared to *k*-means and FCM clustering the AH method produced AIFs that were more in accordance to what was expected. Irreproducible AIFs will lead to unstable quantification of CBF, so the reproducibility of each algorithm was assessed using the same batch of data. The results showed that AH method had perfect reproducibility, whereas *k*-means and FCM methods did not yield stable AIF detection results, which may be related to their sensitivity to the randomly selected initialization parameters. This suggests that AH clustering algorithm is preferable to the two previously reported clustering methods for AIF determination.

In addition, we addressed the computational time requirement of each method. The results demonstrated that the mean execution time was significantly longer with AH method. This may be a problem in clinical practice, but it should be emphasized that this research was conducted using a standard laptop and its limited CPU operating frequency and memory capacity meant that the calculation speed was low compared with a high-performance workstation dedicated to image processing. In our opinion, the disadvantage of a relatively long execution time might be eliminated if the AH-based method was performed on a professional workstation, or at least alleviated, and sometimes it is worthwhile to achieve more accurate AIF.

It should be noted that our research had three main limitations. First, we limited our research to the area around the MCA, which is the most frequently used region. In addition, only a global value was calculated. However, the AIF should be obtained for each pixel, i.e., local AIF values, to reduce the effects of the tracer delay and dispersion on hemodynamic quantification. As reported by Kjølby et al., however, PVE will affect the local AIF selection and quantitative perfusion estimates [39]. Thus, only a few attempts have been made to perform local AIF measurements [4], [23], [32]. For the global AIF, many different deconvolution approaches can be used to reduce the impacts of tracer delay, including time insensitive block-circulant singular value decomposition (cSVD) and nonlinear stochastic regularization (NSR) [4], [21], [33]–[38]. Second, we only focused on evaluating the performance of the three different AIF detection methods and we did not calculate the absolute values of the hemodynamics, such as CBF, CBV, and MTT. In fact, the AIF is only an interim result relative to the final hemodynamic estimates. We did not attempt final hemodynamic quantification because several different deconvolution approaches are available, which have various effects on CBF quantification. Third, all of the participants in the clinical study were healthy and subjects with nervous system diseases were not included, such as acute stroke, arterial stenosis, and other abnormalities.

In conclusion, AH method can obtain absolutely robust AIFs more in line with the expected AIF than the traditional *k*-means and FCM clustering methods. Given its computation complexity, however, it will be necessary to perform AH clustering algorithm using a professional workstation to reduce the runtime.
